# Human–AI collaboration role ambiguity and university teachers’ professional identity: the mediating role of meaningful work

**DOI:** 10.3389/fpsyg.2026.1950622

**Published:** 2026-08-27

**Authors:** Wei Tao, Zheng Li, Aiai Zhang

**Affiliations:** 1 The College of Humanities and Social Sciences, Huazhong Agricultural University, Wuhan, China; 2 School of Languages and Communication Studies, Beijing Jiaotong University, Beijing, China; 3 School of Journalism and Communication, Tsinghua University, Beijing, China

**Keywords:** generative artificial intelligence, meaningful work, professional identity, role ambiguity, university teachers

## Abstract

**Introduction:**

Generative artificial intelligence (GenAI) is changing not only how university teachers perform tasks but also how professional responsibilities are divided between human actors and intelligent systems. Drawing on role theory, meaningful work scholarship, and research on professional identity, this study examined whether meaningful work mediates the longitudinal relationship between role ambiguity in human – AI collaboration and university teachers’ professional identity.

**Methods:**

A three-wave survey of university teachers was conducted at four-week intervals; 1,074 complete matched cases formed the final analytical sample. Role ambiguity, meaningful work, and professional identity were repeatedly measured, and the analyses included formal common-method checks, reliability and validity analyses at every wave, longitudinal measurement-invariance tests, baseline-adjusted hierarchical regressions, and a temporally ordered bootstrap mediation analysis.

**Results:**

Time 1 role ambiguity was associated with lower Time 2 meaningful work (*β* = −0.193, *p* < 0.001; Δ*R*^2^ = 0.031) and lower Time 3 professional identity (*β* = −0.154, *p* < 0.001; Δ*R*^2^ = 0.018). Time 2 meaningful work was positively associated with Time 3 professional identity (*β* = 0.219, *p* < 0.001; Δ*R*^2^ = 0.017). The longitudinal indirect effect was *B* = −0.040 (BootSE = 0.006, 95% CI [−0.052, −0.028]), with a remaining direct association of *B* = −0.111, consistent with partial mediation. These incremental effects were statistically reliable but small.

**Discussion:**

The results suggest that GenAI use frequency alone does not capture the professional consequences of AI-supported academic work; clarity about who does what, who decides, and who remains accountable also matters.

## Graphical abstract

**Figure:**
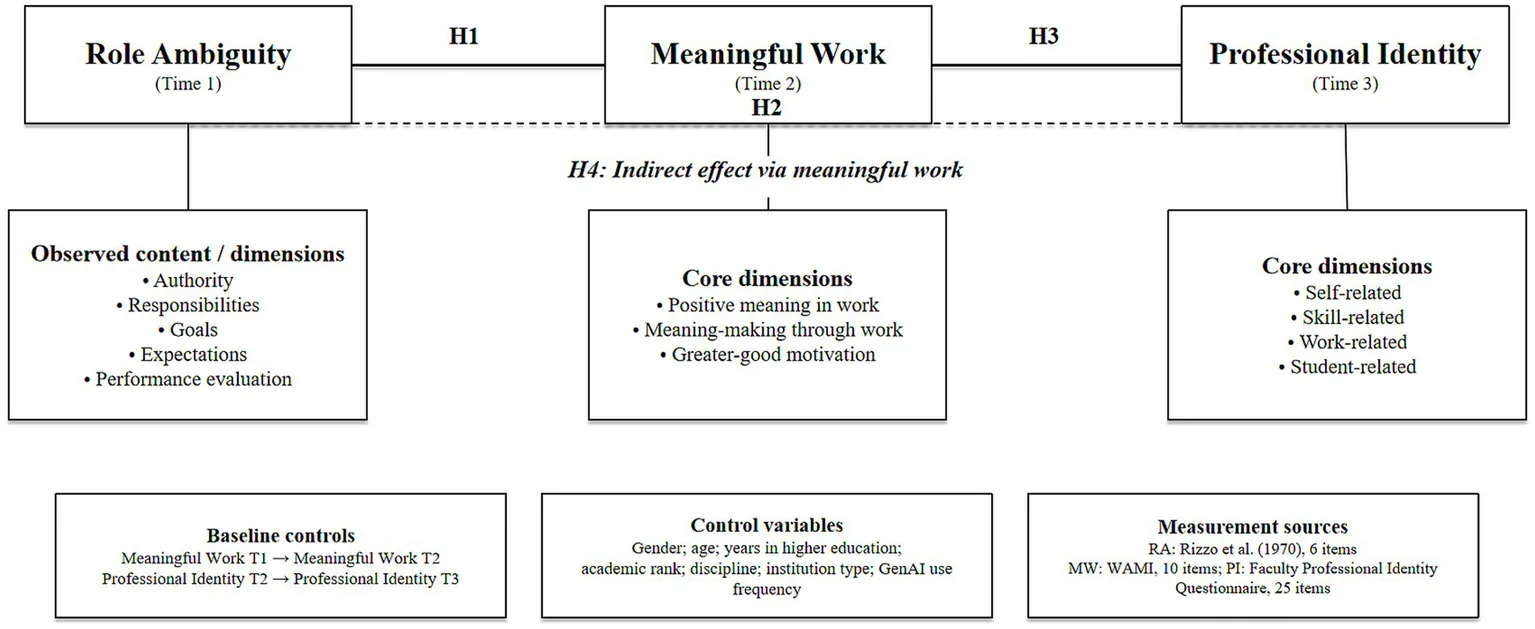

## 1 Introduction

Generative artificial intelligence (GenAI) has rapidly become part of university teaching, assessment, research, writing, student supervision, and administrative work. Faculty research has documented heterogeneous patterns of AI use, self-efficacy, perceived benefits, concerns, and professional-development needs (Mah and Groß, 2024). At the same time, GenAI can participate in activities that have traditionally signaled academic expertise, such as explaining disciplinary content, drafting feedback, suggesting research questions, synthesizing literature, and generating text or code. This creates an automation-augmentation tension: AI may expand teachers’ capabilities while also taking over parts of the work through which professional expertise has historically been demonstrated (Jarrahi, 2018; Raisch and Krakowski, 2021). The resulting question is not only whether faculty use GenAI, but how work is divided when humans and AI jointly contribute to academic tasks.

In this study, human–AI collaboration refers to a sociotechnical work arrangement in which a faculty member and a GenAI system make interdependent contributions to the same work process. This definition draws on research on delegation to agentic information systems and conjoined human-technology agency, which emphasizes the distribution and coordination of tasks and decision rights between human and technological actors (Baird and Maruping, 2021; Murray et al., 2021). GenAI remains different from a human organizational role holder: it does not bear professional or legal responsibility in the same way a faculty member or institution does. Nevertheless, its capacity to generate content and recommendations makes the boundaries of delegation, verification, attribution, and final accountability practically consequential. Recent research likewise shows that the form of human–AI collaboration can shape accountability and job meaningfulness (Sadeghian et al., 2024), and that active collaboration can mitigate losses in ownership and meaning associated with relying on AI at work (Lee et al., 2026).

This perspective exposes a gap in the higher-education literature. Much GenAI research has centered on adoption, frequency of use, attitudes, self-efficacy, academic integrity, or performance. These constructs are important, but they do not indicate whether faculty know which tasks may be delegated, which decisions must remain human, how AI-generated contributions should be disclosed, or who is responsible for an AI-assisted outcome. A teacher can therefore be a frequent and confident GenAI user while still experiencing uncertainty about professional boundaries. Emerging work on teacher identity reinforces the importance of this distinction: AI-enhanced teacher development can create tensions between humanity and technology, continuity and openness, and established authority and a reconstructed collaborator role (Lan, 2024; Li and Zhang, 2026). Yet longitudinal quantitative evidence linking such boundary uncertainty to professional identity remains limited.

A second gap concerns mechanism. Professional identity is not simply an attitude toward a technology; it concerns how individuals understand who they are as members of a profession and whether their everyday work expresses valued professional purposes. Meaningful work is therefore theoretically proximal to identity because it captures the extent to which work feels significant, purposeful, self-expressive, and contributive to a greater good (Rosso et al., 2010; Steger et al., 2012; Martela and Pessi, 2018). Recent human–AI studies show that autonomy, accountability, mental effort, and active involvement affect whether AI-supported work remains meaningful (Sadeghian et al., 2024; Callari and Puppione, 2025; Vodiškar and Ruiner, 2026). This suggests that role-boundary ambiguity may matter for professional identity partly because it changes whether teachers can still experience their work as their own purposeful professional contribution.

Accordingly, this study examines a temporally ordered model in which Time 1 human–AI collaboration role ambiguity is associated with Time 2 meaningful work, which in turn is associated with Time 3 professional identity. The three-wave design includes prior levels of the mediator and outcome and demographic/occupational covariates. The study makes three incremental contributions. First, it extends role theory from ambiguity generated primarily by human role senders to ambiguity arising in the sociotechnical allocation of work between faculty and GenAI. Second, it moves human–AI collaboration research beyond use frequency by focusing on task allocation, decision authority, responsibility, and accountability. Third, it connects human–AI role design to professional identity through meaningful work and tests that mechanism longitudinally while explicitly acknowledging the limits of observational inference.

## 2 Literature review and hypotheses

### 2.1 Role ambiguity in human–AI collaboration

Role theory defines organizational roles as sets of expectations concerning duties, authority, priorities, evaluation criteria, and responsibility (Kahn et al., 1964). Role ambiguity occurs when individuals lack sufficient information about what they are expected to do or how successful role performance will be evaluated (Rizzo et al., 1970). Traditional role ambiguity is therefore typically located in incomplete or inconsistent expectations communicated by supervisors, colleagues, formal job descriptions, or other human role senders. Meta-analytic and measurement research has linked role ambiguity with unfavorable work attitudes and psychological strain because employees cannot confidently direct their effort toward recognizable and valued outcomes (Jackson and Schuler, 1985; Smith et al., 1993).

Human–AI collaboration role ambiguity retains this core logic but changes the source and content of uncertainty. Agentic technologies can now perform parts of tasks, propose actions, and generate outputs rather than merely serving as passive tools (Baird and Maruping, 2021). Conjoined-agency research similarly shows that human and technological agency can be combined in different assisting, augmenting, constraining, and automating configurations (Murray et al., 2021). In academic work, role ambiguity may arise when the boundaries between teachers and AI are unclear. Teachers may be uncertain about which tasks can be delegated to AI, which decisions should remain under human control, and who is responsible for checking the accuracy and appropriateness of AI-generated content. Similar uncertainty may arise over authorship and the evaluation of AI-assisted work (Chan and Hu, 2023). Although these issues are specific to AI-supported work, they can still be understood as manifestations of role ambiguity.

Human–AI collaboration role ambiguity is also distinct from AI anxiety, readiness, acceptance, self-efficacy, and use frequency. Anxiety captures apprehension, readiness and self-efficacy concern perceived capacity, acceptance concerns willingness to use a technology, and frequency captures exposure. None necessarily tells a teacher whether the human–AI division of labor is clear. Mah and Groß (2024), for example, show substantial heterogeneity in faculty AI use and self-efficacy, but role-boundary clarity constitutes a separate question. This distinction is central to the present model: GenAI use frequency is controlled statistically, whereas uncertainty about the human role in AI-supported work is the focal predictor.

Role ambiguity should be negatively associated with meaningful work because meaningfulness depends on being able to connect one’s actions and competencies to valued outcomes. When teachers cannot determine which contributions are genuinely theirs, where their judgment begins and ends, or for which outcomes they remain accountable, the work may provide fewer opportunities to experience agency, ownership, competence, and contribution. In a human–AI experiment, interactive collaboration that kept people involved and accountable was associated with greater job meaningfulness than a supervisory arrangement (Sadeghian et al., 2024). Qualitative research likewise indicates that meaningful work in AI collaboration is shaped by work practices, autonomy, and decision making (Callari and Puppione, 2025), while active collaboration can mitigate reductions in ownership and meaning that occur when workers rely on AI (Lee et al., 2026). These findings support the proposition that unclear human–AI role boundaries are likely to be associated with lower subsequent meaningful work.

*Hypothesis 1*: Greater human-AI collaboration role ambiguity at Time 1 will be associated with lower meaningful work at Time 2 after controlling for Time 1 meaningful work.

Role ambiguity may also be associated with professional identity directly. University teachers’ professional identities are enacted through teaching, research, student supervision, disciplinary membership, institutional service, and public responsibility. If the boundaries of intellectual contribution, authority, authorship, and accountability become unclear, teachers may have greater difficulty sustaining a stable account of what distinguishes their professional role. This issue is salient when tasks once treated as markers of academic expertise are increasingly performed with machine-generated outputs. Recent teacher research documents AI-related identity tensions as well as shifts from an authority-centered identity toward a collaborator role (Lan, 2024; Li and Zhang, 2026). The direction of such identity reconstruction is not necessarily negative; clear and well-designed collaboration may support professional adaptation. The present argument is narrower: persistent ambiguity about the human role should be associated with weaker identity continuity and clarity.

*Hypothesis 2*: Greater human-AI collaboration role ambiguity at Time 1 will be associated with weaker professional identity at Time 3 after controlling for prior professional identity.

### 2.2 Meaningful work and professional identity

Meaningful work refers to work experienced as significant, purposeful, and connected to valued self-development or broader social contribution. Rosso et al. (2010) locate sources of work meaning in the self, other people, the work context, and wider purposes. Martela and Pessi (2018) emphasize positive significance, self-realization, and contribution to a greater good. The Work and Meaning Inventory operationalizes related dimensions as positive meaning in work, meaning-making through work, and greater-good motivation (Steger et al., 2012). Meta-analytic evidence links meaningful work with engagement, commitment, satisfaction, and wellbeing (Allan et al., 2019).

For university teachers, meaningfulness can arise from advancing knowledge, supporting students’ intellectual and personal development, exercising disciplinary and pedagogical judgment, and contributing to society through teaching and research. AI does not necessarily undermine these sources. Recent research instead suggests that meaningfulness depends on how human effort, autonomy, accountability, and machine assistance are configured (Sadeghian et al., 2024; Callari and Puppione, 2025; Vodiškar and Ruiner, 2026). A well-designed arrangement may remove repetitive work and preserve substantive human contribution, whereas an arrangement that makes ownership and responsibility unclear may weaken it.

Professional identity refers to an individual’s understanding of who they are as a member of a profession and of the values, competencies, practices, and relationships that define legitimate professional participation. Teacher identity is dynamic and context dependent rather than a fixed personal characteristic (Beijaard et al., 2004). It develops through expertise, pedagogical competence, relationships with students, professional agency, institutional recognition, and participation in occupational communities (Beijaard et al., 2000; van Lankveld et al., 2017). Identity-construction research further shows that professionals revise their self-understandings when changing work demands disrupt the fit between daily activities and established professional values (Ibarra, 1999; Pratt et al., 2006).

Meaningful work should help sustain professional identity because meaningful experiences affirm that daily activities are legitimate and valuable expressions of the professional self. The faculty professional-identity measure used here includes self-related, skill-related, work-related, and student-related dimensions (Abu-Alruz and Khasawneh, 2013). Positive meaning can reinforce the value of being a university teacher; meaning-making can connect work experiences with a coherent professional self; and greater-good motivation can connect professional identity with students, scholarship, and social contribution. Thus, after accounting for prior identity, teachers who continue to experience their work as meaningful should be more likely to report stronger subsequent professional identity.

*Hypothesis 3*: Greater meaningful work at Time 2 will be associated with stronger professional identity at Time 3 after controlling for prior professional identity.

### 2.3 The mediating role of meaningful work

The proposed mediation combines two theoretically distinct steps. In the first step, human–AI role ambiguity is expected to erode meaningful work by making agency, ownership, responsibility, and contribution harder to locate. This argument concerns the design and interpretation of the work itself: if teachers cannot tell which parts of an AI-assisted outcome reflect their expertise or where their responsibility lies, the connection between personal action and valued professional purpose becomes less clear. Evidence that accountability and active involvement matter for meaningfulness in human–AI collaboration supports this first link (Sadeghian et al., 2024; Lee et al., 2026).

In the second step, meaningful work is expected to support professional identity because it provides experiential evidence that the occupation remains significant and self-defining. When teaching, research, and supervision still feel purposeful and socially contributive, faculty can integrate new technology into an identity that remains centered on professional expertise and responsibility. Conversely, if AI-supported work feels less personally attributable or significant, the identity of ‘university teacher’ may become less central or less valued. Recent qualitative studies of teachers in AI-enhanced environments show that professional identity is actively negotiated as educators reconcile technological change with valued professional roles (Lan, 2024; Li and Zhang, 2026).

Meaningful work was selected as the focal mediator rather than technology anxiety, job insecurity, workload, or self-efficacy because it is conceptually closer to the identity outcome: it directly captures whether work remains a significant expression of self and social contribution. This choice does not imply that alternative mechanisms are unimportant. Indeed, autonomy, self-efficacy, recognition, technology anxiety, and perceived replacement risk are plausible parallel mechanisms and are treated as directions for future research. The expectation here is therefore partial rather than complete mediation.

**Figure 1:**
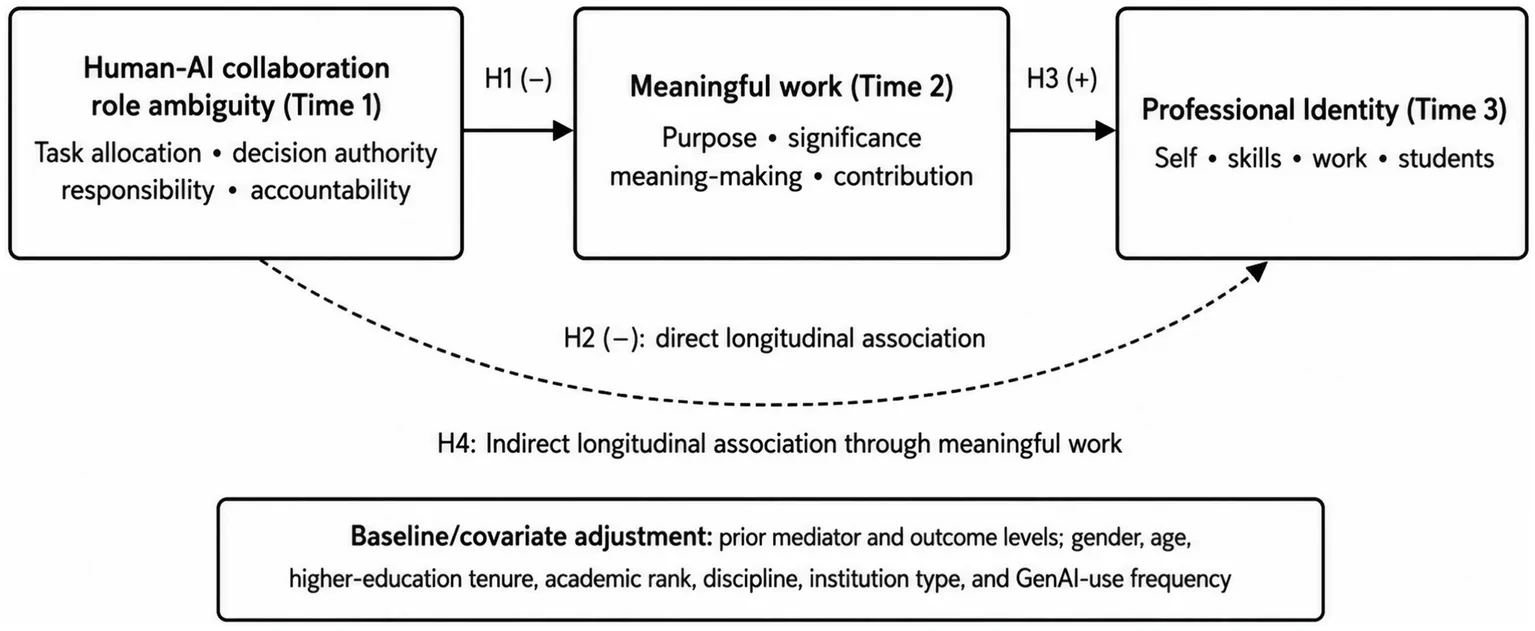
Consolidated conceptual and longitudinal analytical framework.

The temporal model also improves on cross-sectional mediation by separating the predictor, mediator, and outcome and controlling prior levels. Cross-sectional mediation can yield biased conclusions when temporal ordering and autoregressive stability are ignored (Cole and Maxwell, 2003; Maxwell and Cole, 2007). Our primary mediation specification predicts Time 2 meaningful work from Time 1 role ambiguity while controlling Time 1 meaningful work and predicts Time 3 professional identity from Time 2 meaningful work and Time 1 role ambiguity while controlling Time 2 professional identity and Time 1 meaningful work (Figure 1).

*Hypothesis 4*: Meaningful work at Time 2 will statistically mediate the longitudinal association between Time 1 human-AI collaboration role ambiguity and Time 3 professional identity.

## 3 Materials and methods

### 3.1 Study design, sampling, and measurement interval

The study used a three-wave, time-lagged survey design. The focal constructs were measured at Time 1 (week 0), Time 2 (week 4), and Time 3 (week 8). The primary temporal sequence specified Time 1 role ambiguity as the predictor, Time 2 meaningful work as the mediator, and Time 3 professional identity as the outcome, with prior mediator/outcome levels included as covariates. This design strengthens temporal ordering relative to a single-wave survey.

A non-probability, multi-institutional voluntary-response sampling strategy was used among full-time faculty employed by higher-education institutions in mainland China. Recruitment invitations were circulated electronically through faculty-facing academic and professional communication networks associated with multiple higher-education institutions. Eligibility required current full-time employment as university faculty, adult status, and work-related exposure to or use of GenAI in teaching, research, student supervision, academic writing/data work, communication, or administrative service. University names, province identifiers, and channel-level invitation records were not included in the de-identified analytical dataset. The sample is therefore described as multi-institutional rather than nationally representative. Its composition is reported descriptively by academic rank, discipline, institution type, age, career tenure, gender, and GenAI-use frequency.

The four-week interval was chosen as a pragmatic compromise for a rapidly changing work context. Perceptions of role boundaries and meaningfulness can change over weeks as faculty repeatedly use GenAI and encounter evolving local norms, while temporal separation reduces same-occasion response carryover. Because panel estimates can vary with lag length (Dormann and Griffin, 2015), the four-week interval is treated as a design choice rather than an assumption about the optimal causal timescale (Table 1).

**Table 1:** Three-wave data-collection schedule.

Wave	Timing	Core constructs	Additional content
Time 1	Week 0	Role ambiguity; meaningful work; professional identity	Eligibility; demographics; GenAI use
Time 2	Week 4	Role ambiguity; meaningful work; professional identity	Repeated work-context measures
Time 3	Week 8	Role ambiguity; meaningful work; professional identity	Repeated work-context measures

### 3.2 Procedure, participant protection, and sample formation

The longitudinal records indicate that 1,300 eligible respondents participated at Time 1. Of these respondents, 1,178 were observed again at Time 2, and 1,074 had matched records at Time 3. The final analytical sample therefore consisted of 1,074 participants with complete observations across all three measurement occasions (Table 2).

Before participation, respondents were informed of the study purpose, survey procedures, voluntary nature of participation, intended academic use of the responses, and confidentiality protections. Proceeding to and submitting the questionnaire after reading this information indicated informed consent. Anonymous study IDs were used solely for longitudinal matching, and direct personal identifiers were not included in the analytical dataset.

Before the statistical analyses, the final three-wave matched file was checked for duplicate study IDs, missing responses on the focal measures, values outside the permitted 1–5 response range, and evident inconsistencies in the retained demographic information. No additional cases were removed at this stage. The resulting analytical sample comprised 1,074 complete three-wave cases. Table 2 summarizes the sample formation and analysis-stage screening process.

**Table 2:** Sample formation, retention, and data screening.

Stage/criterion	Retained *N*	Change from preceding stage	Note
Time 1 eligible respondents	1,300	–	Eligible full-time university faculty with work-related GenAI exposure/use
Time 2 retained respondents	1,178	−122	122 Time 1 respondents were not observed at Time 2
Time 3 retained/complete matched respondents	1,074	−104	A further 104 respondents were not observed at Time 3; cumulative non-retention from Time 1 = 226
Analysis-stage data screening	1,074	0	No additional exclusions
Final analytical sample	1,074	–	Complete three-wave matched cases used in the analyses

A total of 226 Time 1 respondents were not represented in the final three-wave matched sample.

### 3.3 Measures

Role ambiguity. Role ambiguity was measured at each wave with the six-item scale developed by Rizzo et al. (1970). The questionnaire instructions explicitly asked respondents to answer with reference to their university work when using or collaborating with GenAI across teaching, research, student supervision, and academic service. The items assess clarity concerning authority, responsibilities, goals, expectations, task organization, and performance-related requirements. Responses were recorded on a five-point scale and oriented so that higher mean scores indicated greater ambiguity. In the human–AI context, authority maps onto decision rights, responsibility maps onto accountability and verification duties, goals/expectations map onto what work should remain human versus be delegated, and task organization maps onto division of labor. The scale remains a contextualized application of the established Rizzo measure rather than a newly validated human–AI-specific scale; this measurement boundary is revisited in Section 5.2.

Meaningful work. Meaningful work was measured at each wave with the 10-item Work and Meaning Inventory (WAMI; Steger et al., 2012), covering positive meaning in work, meaning-making through work, and greater-good motivation. Items were averaged on the common 1–5 metric, with higher scores indicating stronger perceived significance, purpose, and social contribution.

Professional identity. Professional identity was measured at each wave with the 25-item Professional Identity Questionnaire for Faculty Members (Abu-Alruz and Khasawneh, 2013), which includes self-related, skill-related, work-related, and student-related content. Because professional identity was modeled as an overall outcome, all items were averaged to form a 1–5 score, with higher values indicating stronger identification with the values, competencies, responsibilities, and relationships associated with university teaching.

Control variables. All regression models included gender, age, years employed in higher education, academic rank, discipline, institution type, and work-related GenAI-use frequency. GenAI use frequency was controlled specifically to distinguish exposure/use from role-boundary ambiguity. Categorical variables were dummy coded; the reference categories were women, lecturer, education discipline, teaching/applied institution, and GenAI use 1–2 times per week. Age and years in higher education were standardized for the regression analyses.

### 3.4 Analytical strategy

Analyses were conducted on item-level data with two-tailed tests and *α* = 0.05. Descriptive statistics and Pearson correlations were calculated for all three constructs at all three waves. Internal consistency was evaluated separately at each wave using Cronbach’s *α* and composite reliability (CR), and convergent validity was evaluated using standardized factor loadings and average variance extracted (AVE). Discriminant validity was examined with three-factor confirmatory factor analysis (CFA), the Fornell–Larcker criterion (Fornell and Larcker, 1981), and heterotrait-monotrait (HTMT) ratios (Henseler et al., 2015).

To address common-method concerns formally, Harman’s unrotated single-factor diagnostic was calculated separately at each wave using all focal items. Because this diagnostic is limited, it was interpreted together with a one-factor CFA compared with the theorized three-factor measurement model and with the procedural remedies of anonymity and temporal separation (Podsakoff et al., 2003). The analysis is therefore a diagnostic of severe single-source dominance, not proof that common-method variance is absent.

Longitudinal measurement invariance was tested separately for role ambiguity, meaningful work, and professional identity using a sequence of configural, metric, and scalar CFA models across the three waves. Same-item residuals were allowed to correlate across waves. Changes in CFI and RMSEA were considered alongside overall fit, following common recommendations for invariance evaluation (Cheung and Rensvold, 2002; Chen, 2007). Configural and metric invariance are particularly relevant for comparing longitudinal structural associations; scalar invariance is required for stronger latent-mean interpretations.

Hierarchical regression analyses were estimated sequentially for the two dependent variables. For Time 2 meaningful work, Model MW-1 included all controls and Time 1 meaningful work, and Model MW-2 added Time 1 role ambiguity. For Time 3 professional identity, Model PI-1 included all controls, Time 2 professional identity, and Time 1 meaningful work; Model PI-2 added Time 1 role ambiguity; and Model PI-3 added Time 2 meaningful work. Continuous focal variables were standardized before these regressions; heteroskedasticity-consistent HC3 standard errors were used. *R*^2^, Δ*R*^2^, Δ*F*, and the significance of each model comparison are reported together with the full coefficients for every control variable.

The longitudinal mediation analysis followed the temporal adjustment structure of the hierarchical models (Hayes, 2022). The a equation predicted Time 2 meaningful work from Time 1 role ambiguity while controlling Time 1 meaningful work and the demographic/occupational controls. The outcome equation predicted Time 3 professional identity from Time 2 meaningful work and Time 1 role ambiguity while controlling Time 2 professional identity, Time 1 meaningful work, and the same controls. The indirect effect was estimated with 5,000 bootstrap resamples. As robustness checks, an alternative symmetrical-covariate specification additionally included Time 2 professional identity in the mediator equation, and the focal regressions were re-estimated after omitting age or years in higher education because those controls were highly correlated.

## 4 Results

### 4.1 Sample characteristics and three-wave descriptive statistics

The final analytical sample comprised 1,074 complete three-wave records. Men represented 51.3% and women 48.7% of the sample. Mean age was 43.01 years (SD = 8.39), and mean employment duration in higher education was 14.05 years (SD = 8.54). The sample included lecturers, associate professors, and professors; six broad disciplinary groups; three institution types; and three regular GenAI-use categories. The sample was heterogeneous across these characteristics, although the non-probability design and absence of detailed identifiers limit claims of national representativeness (Table 3).

**Table 3:** Characteristics of the analytical sample (*N* = 1,074).

Characteristic	Category/statistic	Value
Age, years	*M* (SD)	43.01 (8.39)
Higher-education employment, years	*M* (SD)	14.05 (8.54)
Gender	Men	551 (51.3%)
	Women	523 (48.7%)
Academic rank	Professor	373 (34.7%)
	Associate professor	373 (34.7%)
	Lecturer	328 (30.5%)
Discipline	Engineering	199 (18.5%)
	Medicine	186 (17.3%)
	Science	176 (16.4%)
	Humanities and social sciences	173 (16.1%)
	Management	170 (15.8%)
	Education	170 (15.8%)
Institution type	Comprehensive	451 (42.0%)
	Research-intensive	375 (34.9%)
	Teaching/applied	248 (23.1%)
GenAI use frequency	Almost daily	362 (33.7%)
	1–2 times per week	361 (33.6%)
	3–5 times per week	351 (32.7%)

Percentages may not sum to 100 because of rounding.

**Figure 2:**
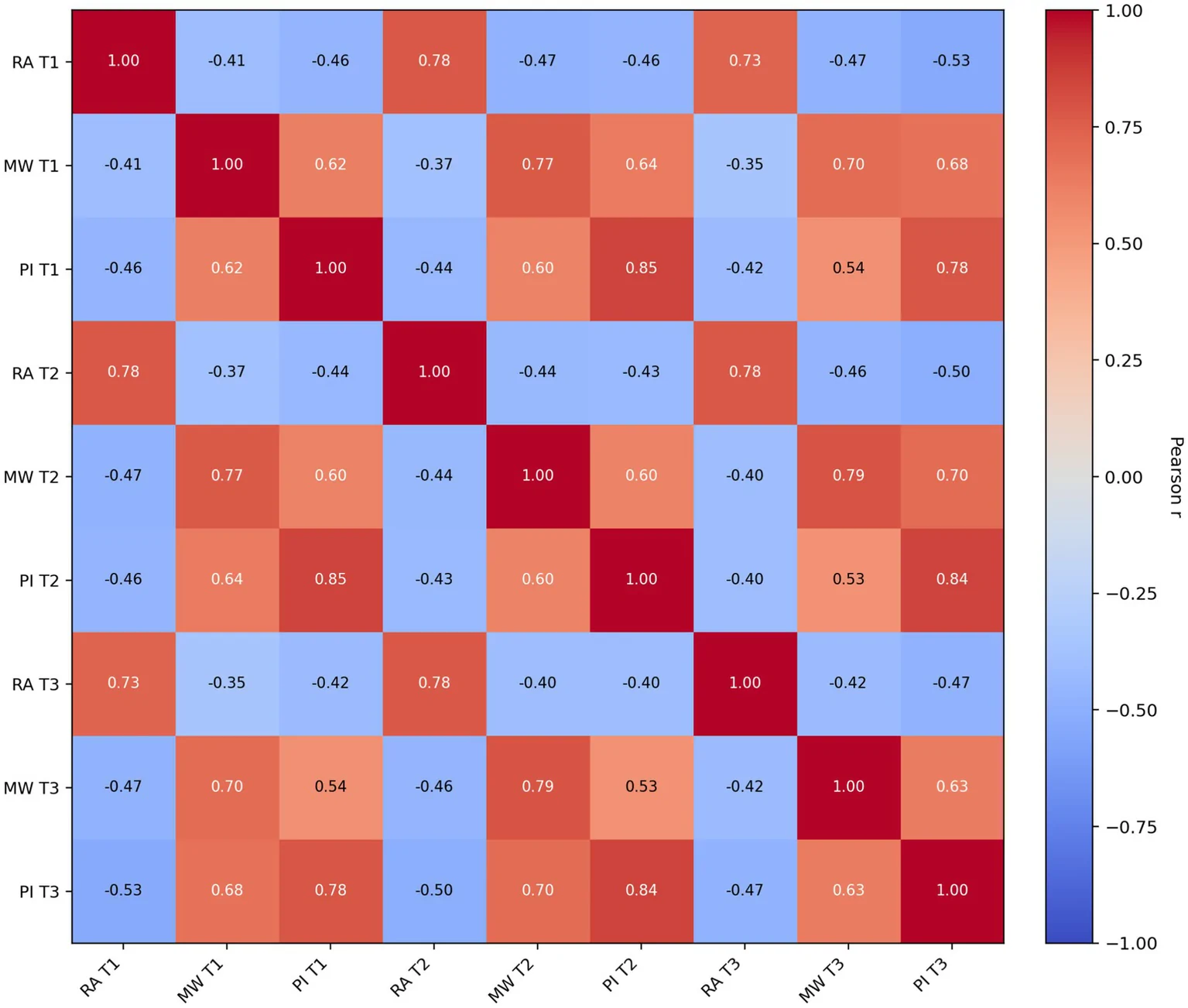
Correlations among the three focal constructs across all three measurement waves.

Across waves, role ambiguity means ranged from 2.791 to 2.923, meaningful work means from 3.241 to 3.327, and professional identity means from 3.327 to 3.394. Table 4 reports the complete three-wave correlation matrix. All off-diagonal correlations were statistically significant at *p* < 0.001. Of particular relevance, Time 1 role ambiguity correlated with Time 2 meaningful work at *r* = −0.473 and Time 3 professional identity at *r* = −0.529, while Time 2 meaningful work correlated with Time 3 professional identity at *r* = 0.699. Longitudinal stability was substantial, including *r* = 0.845 between Time 2 and Time 3 professional identity, underscoring the stringency of detecting incremental predictors after prior levels were controlled (Figure 2).

**Table 4:** Complete three-wave descriptive statistics and Pearson correlations.

Variable	M	SD	1	2	3	4	5	6	7	8	9
1. RA T1	2.846	0.982	–								
2. MW T1	3.241	0.966	−0.406***	–							
3. PI T1	3.371	0.923	−0.458***	0.619***	–						
4. RA T2	2.791	0.996	0.777***	−0.372***	−0.440***	–					
5. MW T2	3.301	0.953	−0.473***	0.772***	0.605***	−0.436***	–				
6. PI T2	3.327	0.926	−0.459***	0.641***	0.851***	−0.428***	0.603***	–			
7. RA T3	2.923	0.978	0.729***	−0.349***	−0.421***	0.778***	−0.404***	−0.401***	–		
8. MW T3	3.327	0.982	−0.473***	0.696***	0.541***	−0.460***	0.794***	0.532***	−0.421***	–	
9. PI T3	3.394	0.919	−0.529***	0.679***	0.784***	−0.503***	0.699***	0.845***	−0.474***	0.628***	–

RA, role ambiguity; MW, meaningful work; PI, professional identity; T1–T3, measurement waves. ***p < 0.001. Correlations are based on *N* = 1,074 complete cases.

### 4.2 Reliability, construct validity, common-method diagnostics, and measurement invariance

Reliability and convergent-validity statistics were examined at every wave (Table 5). Cronbach’s *α* ranged from 0.858 to 0.961 and CR from 0.858 to 0.961. Standardized loading ranges were 0.687–0.756 for role ambiguity, 0.680–0.765 for meaningful work, and 0.660–0.749 for professional identity across the three waves. AVE exceeded 0.50 for role ambiguity and meaningful work at most waves. Professional identity showed AVE values of 0.489, 0.494, and 0.492, slightly below the conventional 0.50 guideline. Given this small shortfall, its convergent validity was considered marginal, although CR remained 0.960–0.961 and all standardized loadings were at least 0.660. The pattern suggests strong internal consistency alongside a modest limitation in convergent validity.

**Table 5:** Reliability and convergent validity at each measurement wave.

Construct	Wave	Items	Cronbach *α*	CR	AVE	Loading range
Role ambiguity	T1	6	0.859	0.859	0.504	0.695–0.738
Meaningful work	T1	10	0.912	0.912	0.511	0.682–0.765
Professional identity	T1	25	0.960	0.960	0.489	0.663–0.719
Role ambiguity	T2	6	0.863	0.863	0.513	0.698–0.756
Meaningful work	T2	10	0.912	0.912	0.509	0.680–0.749
Professional identity	T2	25	0.961	0.961	0.494	0.668–0.741
Role ambiguity	T3	6	0.858	0.858	0.501	0.687–0.732
Meaningful work	T3	10	0.919	0.919	0.531	0.703–0.753
Professional identity	T3	25	0.960	0.960	0.492	0.660–0.749

CR, composite reliability; AVE, average variance extracted. All focal items were coded on a five-point scale.

The hypothesized three-factor CFA model fitted substantially better than a one-factor model at every wave (Table 6). The three-factor CFI values were 0.999, 0.997, and 0.996, with RMSEA values from 0.006 to 0.010 and SRMR values from 0.018 to 0.019. By contrast, one-factor CFI values ranged from 0.803 to 0.814 and RMSEA from 0.071 to 0.074. HTMT ratios were also below 0.85 at all waves (RA-MW = 0.458–0.492; RA-PI = 0.470–0.522; MW-PI = 0.644–0.668). Together, these findings support empirical distinctiveness among role ambiguity, meaningful work, and professional identity.

**Table 6:** Confirmatory factor analyses and common-method diagnostics by wave.

Wave	Model	*χ* ^2^	df	CFI	TLI	RMSEA	SRMR
T1	Three-factor	804.52	776	0.999	0.999	0.006	0.019
T1	One-factor	5,021.52	779	0.814	0.805	0.071	0.076
T2	Three-factor	845.25	776	0.997	0.997	0.009	0.019
T2	One-factor	5,333.13	779	0.803	0.793	0.074	0.081
T3	Three-factor	864.37	776	0.996	0.996	0.010	0.018
T3	One-factor	5,166.15	779	0.813	0.803	0.072	0.075

Harman’s unrotated first factor accounted for 40.18% (T1), 40.04% (T2), and 40.87% (T3) of item variance.

Harman’s unrotated first factor accounted for 40.18, 40.04, and 40.87% of item variance at Times 1–3, respectively. No single factor therefore accounted for a majority of the variance. These results were considered together with the substantially poorer one-factor CFA models and the procedural safeguards of anonymity and temporal separation. Common-source bias cannot be excluded and remains a limitation of the self-report design (Table 7).

**Table 7:** Longitudinal measurement invariance across the three waves.

Construct	Model	*χ* ^2^	df	CFI	RMSEA	ΔCFI	ΔRMSEA
Role ambiguity	Configural	116.12	114	1.000	0.004		
Role ambiguity	Metric	132.64	126	0.999	0.007	−0.000	+0.003
Role ambiguity	Scalar	347.89	136	0.977	0.038	−0.022	+0.031
Meaningful work	Configural	459.14	372	0.995	0.015		
Meaningful work	Metric	505.81	392	0.994	0.016	−0.002	+0.002
Meaningful work	Scalar	990.31	410	0.967	0.036	−0.026	+0.020
Professional identity	Configural	2,832.08	2,622	0.996	0.009		
Professional identity	Metric	2,901.36	2,672	0.995	0.009	−0.000	+0.000
Professional identity	Scalar	3,818.27	2,720	0.977	0.019	−0.018	+0.010

Same-item residuals were allowed to correlate across waves. Changes are relative to the immediately less constrained model.

Configural and metric invariance were supported for all three constructs: the metric models showed very small changes in CFI (|ΔCFI| ≤ 0.002) and RMSEA (ΔRMSEA ≤ 0.003). Full scalar invariance was not supported, however, because adding intercept constraints reduced CFI by 0.022 for role ambiguity, 0.026 for meaningful work, and 0.018 for professional identity. Accordingly, the longitudinal interpretation focuses on structural associations at the metric level rather than latent mean change.

### 4.3 Hierarchical regression analyses

The hierarchical regressions were estimated sequentially and separately for the two dependent variables. Table 8 shows the prediction of Time 2 meaningful work. The baseline model containing controls and Time 1 meaningful work explained 60.0% of the variance. Adding Time 1 role ambiguity increased *R*^2^ by 0.031, Δ*F*(1, 1,057) = 88.35, *p* < 0.001, and role ambiguity was negatively associated with Time 2 meaningful work (*β* = −0.193, HC3 SE = 0.021, *p* < 0.001). Hypothesis 1 was supported. The corresponding incremental *f*^2^ was 0.084, which is small by conventional benchmarks.

**Table 8:** Hierarchical regression predicting Time 2 meaningful work.

Predictor/statistic	MW-1: baseline	MW-2: + RA T1
Male (vs female)	−0.037 (0.039)	−0.029 (0.038)
Age	−0.022 (0.047)	−0.004 (0.046)
Years in higher education	0.032 (0.049)	0.011 (0.048)
Associate professor (vs lecturer)	0.046 (0.054)	0.066 (0.052)
Professor (vs lecturer)	0.121 (0.071)	0.139* (0.069)
Engineering (vs education)	0.054 (0.071)	0.049 (0.069)
Medicine (vs education)	0.015 (0.071)	0.020 (0.069)
Science (vs education)	0.049 (0.073)	0.053 (0.070)
Humanities/social sciences (vs education)	0.042 (0.070)	0.025 (0.067)
Management (vs education)	0.057 (0.074)	0.079 (0.072)
Comprehensive (vs teaching/applied)	0.014 (0.051)	0.022 (0.050)
Research-intensive (vs teaching/applied)	−0.002 (0.052)	0.001 (0.051)
GenAI 3–5 times/week (vs 1–2/week)	0.039 (0.048)	0.039 (0.046)
GenAI Almost daily (vs 1–2/week)	−0.008 (0.047)	0.009 (0.046)
MW T1	0.765*** (0.018)	0.686*** (0.020)
RA T1	–	−0.193*** (0.021)
*R* ^2^	0.600	0.631
Δ*R*^2^	–	0.031
Δ*F*	–	88.35***

Entries are standardized-regression coefficients with HC3 robust SE in parentheses. Continuous variables were standardized; categorical variables are dummy-coded standardized-outcome contrasts. Reference categories: women, lecturer, education, teaching/applied institution, and GenAI 1–2 times/week. *p < 0.05; **p < 0.01; ***p < 0.001.

Table 9 shows the prediction of Time 3 professional identity. The baseline model containing controls, Time 2 professional identity, and Time 1 meaningful work explained 74.9% of the variance. Adding Time 1 role ambiguity increased *R*^2^ by 0.018, Δ*F*(1, 1,056) = 81.34, *p* < 0.001; role ambiguity was negatively associated with Time 3 professional identity (*β* = −0.154, HC3 SE = 0.017, *p* < 0.001), supporting Hypothesis 2. Adding Time 2 meaningful work increased *R*^2^ by a further 0.017, Δ*F*(1, 1,055) = 84.88, *p* < 0.001. Meaningful work was positively associated with Time 3 professional identity (*β* = 0.219, HC3 SE = 0.025, *p* < 0.001), supporting Hypothesis 3, while the role-ambiguity coefficient remained negative (*β* = −0.118, *p* < 0.001). The incremental *f*^2^ values were 0.077 for role ambiguity and 0.080 for meaningful work, again indicating small effects after strong autoregressive and covariate adjustment.

**Table 9:** Hierarchical regression predicting Time 3 professional identity.

Predictor/statistic	PI-1: baseline	PI-2: + RA T1	PI-3: + MW T2
Male (vs female)	0.005 (0.031)	0.011 (0.030)	0.017 (0.029)
Age	−0.080* (0.039)	−0.065 (0.037)	−0.063 (0.036)
Years in higher education	0.105** (0.040)	0.088* (0.038)	0.085* (0.037)
Associate professor (vs lecturer)	−0.003 (0.043)	0.024 (0.041)	0.015 (0.040)
Professor (vs lecturer)	0.007 (0.054)	0.041 (0.053)	0.020 (0.051)
Engineering (vs education)	−0.019 (0.053)	−0.021 (0.051)	−0.031 (0.049)
Medicine (vs education)	−0.015 (0.054)	−0.018 (0.052)	−0.025 (0.049)
Science (vs education)	−0.021 (0.057)	−0.023 (0.055)	−0.037 (0.053)
Humanities/social sciences (vs education)	−0.066 (0.056)	−0.079 (0.053)	−0.085 (0.051)
Management (vs education)	−0.014 (0.058)	0.009 (0.055)	−0.005 (0.052)
Comprehensive (vs teaching/applied)	0.072 (0.040)	0.074 (0.039)	0.068 (0.037)
Research-intensive (vs teaching/applied)	0.058 (0.042)	0.063 (0.041)	0.064 (0.039)
GenAI 3–5 times/week (vs 1–2/week)	−0.015 (0.039)	−0.014 (0.037)	−0.022 (0.036)
GenAI Almost daily (vs 1–2/week)	0.002 (0.038)	0.012 (0.037)	0.009 (0.035)
PI T2	0.692*** (0.020)	0.636*** (0.021)	0.610*** (0.021)
MW T1	0.232*** (0.022)	0.204*** (0.021)	0.067* (0.026)
RA T1	–	−0.154*** (0.017)	−0.118*** (0.017)
MW T2	–	–	0.219*** (0.025)
*R* ^2^	0.749	0.767	0.784
Δ*R*^2^	–	0.018	0.017
Δ*F*	–	81.34***	84.88***

Entries are standardized-regression coefficients with HC3 robust SE in parentheses. Continuous variables were standardized; categorical variables are dummy-coded standardized-outcome contrasts. Reference categories are as in Table 8. *p < 0.05; **p < 0.01; ***p < 0.001.

Multicollinearity diagnostics indicated a maximum VIF of 7.47, driven primarily by age and years employed in higher education, which were strongly correlated (*r* = 0.920). This represents moderate-to-high collinearity among two control variables and can inflate their individual standard errors. Sensitivity analyses did not materially change the focal results: after omitting age, the focal coefficients were *β* = −0.193 (RA → MW), *β* = −0.119 (RA → PI in the final model), and *β* = 0.219 (MW → PI); after omitting years in higher education, the corresponding values were −0.193, −0.120, and 0.219. The focal conclusions were unchanged.

### 4.4 Longitudinal mediation and robustness analyses

The longitudinal mediation analysis used the same temporal adjustment logic as the hierarchical regressions. In the mediator equation, Time 1 role ambiguity predicted Time 2 meaningful work after Time 1 meaningful work and controls (*a* = *B* = −0.187; standardized *β* = −0.193, *p* < 0.001). In the outcome equation, Time 2 meaningful work predicted Time 3 professional identity after Time 2 professional identity, Time 1 meaningful work, Time 1 role ambiguity, and controls (*b* = *B* = 0.211; standardized *β* = 0.219, *p* < 0.001). The direct effect of role ambiguity remained significant (*c*′ = *B* = −0.111; standardized *β* = −0.118, *p* < 0.001). The 5,000-resample indirect effect was *B* = −0.040 (BootSE = 0.006, 95% CI [−0.052, −0.028]), supporting Hypothesis 4 and a partial indirect association. The corresponding standardized indirect effect was approximately −0.042 (Table 10).

**Table 10:** Longitudinal mediation and sensitivity analysis.

Effect/path	*B*	Standardized	BootSE	95% bootstrap CI	*p*/conclusion
*a*: RA T1 → MW T2	−0.187	−0.193	–	–	< 0.001
*b*: MW T2 → PI T3	0.211	0.219	–	–	< 0.001
*c*′: direct RA T1 → PI T3	−0.111	−0.118	–	–	< 0.001
*c*: total RA T1 → PI T3	−0.144	−0.154	–	–	< 0.001
Indirect *a* × *b*	−0.040	−0.042	0.006	[−0.052, −0.028]	Supported
Sensitivity indirect effect	−0.034	–	0.006	[−0.046, −0.023]	Same conclusion

Primary *a* equation: MW T2 ~ RA T1 + MW T1 + demographic/occupational controls. Primary outcome equation: PI T3 ~ MW T2 + RA T1 + PI T2 + MW T1 + controls. Indirect effect used 5,000 bootstrap samples.

The indirect effect was statistically reliable but modest, and the remaining direct association indicates that meaningful work represents only one pathway linking role ambiguity with professional identity. Professional autonomy, uncertainty about expertise, recognition, job insecurity, technology anxiety, and perceived replacement risk are plausible alternatives for future testing.

## 5 Discussion and conclusion

### 5.1 Main findings and interpretation

The study yielded a consistent longitudinal pattern: greater Time 1 human–AI collaboration role ambiguity was associated with lower Time 2 meaningful work and weaker Time 3 professional identity after substantial prior-level and demographic/occupational adjustment, while Time 2 meaningful work was positively associated with subsequent professional identity. The bootstrap analysis was consistent with a partial indirect pathway through meaningful work. These results remained evident after accounting for GenAI-use frequency, which itself did not emerge as the focal explanatory factor. The findings therefore support a distinction between how often faculty use GenAI and how clearly the human–AI division of labor is organized.

The role-ambiguity finding converges with human–AI research emphasizing delegation, accountability, and conjoined agency. Baird and Maruping (2021) argue that agentic information systems require explicit theorizing about delegation and coordination, while Murray et al. (2021) show that human and technological agency can be combined in qualitatively different forms. Our results add an occupational-identity implication: when the allocation of authority and responsibility remains unclear, the issue is not only workflow efficiency but also how professionals experience their contribution and identity.

The meaningful-work mechanism also aligns with recent empirical evidence. Sadeghian et al. (2024) found that human–AI interaction arrangements preserving involvement and accountability were associated with greater job meaningfulness. Callari and Puppione (2025) similarly show that meaningful work in AI collaboration is shaped through autonomy and decision-making practices, and Vodiškar and Ruiner (2026) emphasize the role of human mental effort in work meaningfulness. Most directly, Lee et al. (2026) report that relying on AI can reduce ownership and meaning whereas active collaboration mitigates those effects. Our findings extend this logic to university faculty and connect meaningful work with a downstream professional-identity outcome.

The professional-identity results are also consistent with recent teacher studies documenting AI-related identity negotiation. Lan (2024) identifies tensions involving humanity versus technology and continuity versus openness in AI-enhanced teacher training. Li and Zhang (2026) describe a shift from authority crisis toward collaborator agency among university teachers. Our results do not imply that AI inevitably weakens identity; rather, they identify role ambiguity as one condition under which identity may be less strongly maintained. This boundary is important because well-specified human–AI collaboration could plausibly support rather than threaten professional identity.

Effect size matters. The focal predictors added approximately 1.7–3.1 percentage points of explained variance after models already included strong prior levels, and the associated incremental *f*^2^ values were about 0.08. These effects are small. Their practical significance therefore lies less in predicting dramatic short-term identity change and more in identifying a potentially cumulative, modifiable feature of work design. Because professional identity was highly stable from Time 2 to Time 3 (*r* = 0.845), even small incremental associations warrant attention, but they should not be overstated.

### 5.2 Theoretical contributions and boundary conditions

This study extends role-ambiguity research by showing how a familiar organizational construct takes on additional sociotechnical content in AI-supported professional work. In academic settings, ambiguity may concern not only expectations and responsibilities, but also task delegation, decision authority, verification, authorship, and accountability between faculty and AI systems.

The findings also suggest that human–AI collaboration cannot be understood from use frequency alone. Faculty may use GenAI regularly while remaining uncertain about professional boundaries. This shifts attention from simple exposure to AI toward how collaboration is organized and how responsibilities are understood.

Meaningful work further helps explain how these role boundaries may relate to professional identity. When faculty are less certain about their contribution and responsibility in AI-supported work, their work may feel less clearly connected to professional purpose and expertise. The partial mediation result suggests that meaningful work is one relevant pathway rather than a complete explanation.

These conclusions remain context-dependent. The sample consists of university faculty in mainland China, and the relationships may vary across national systems, disciplines, career stages, institutional policies, and AI-use scenarios. In addition, the role-ambiguity measure was adapted from a general scale rather than designed specifically for human–AI collaboration, suggesting a need for further replication and measurement refinement.

### 5.3 Practical implications

The evidence supports task-specific role clarification rather than broad encouragement or prohibition of GenAI. Universities should state what AI may assist with, which decisions require human professional judgment, who verifies outputs, when AI contributions must be disclosed, and who remains accountable. Because the observed effects are small and observational, these recommendations are framed as low-risk governance and work-design measures (Table 11).

**Table 11:** Scenario-specific human–AI role-boundary recommendations.

Scenario	AI-supportable functions	Faculty-reserved responsibility	Recommended boundary rule
Teaching and assessment	Draft examples/materials; formative feedback suggestions; item generation	Learning objectives; pedagogical judgment; final grading; fairness and accuracy	Teacher verifies and signs off; disclose material AI use where required; AI does not make unreviewed consequential assessment decisions
Research and academic writing	Ideation; summarization; coding/data support; language editing	Research questions; interpretation; source verification; authorship; ethics; data confidentiality	AI is not treated as accountable author; human authors verify sources/claims and disclose use according to journal/institution policy
Student supervision	Resource suggestions; planning prompts; routine communication drafts	Mentoring relationship; sensitive advice; progression decisions; ethical judgment	AI remains advisory; faculty retains final responsibility for individualized and consequential guidance
Administration and service	Drafting; scheduling; summarizing non-sensitive materials	Decisions affecting staff/students; interpretation of policy; accountability for errors	Human decision owner is named; sensitive data and high-impact decisions require explicit safeguards and review

Training should likewise go beyond prompt engineering. Faculty development should include authorship and disclosure norms, verification responsibilities, limits of AI decision making, data confidentiality, and reflection on which parts of academic work are identity-defining and should remain substantively human-led. This is consistent with evidence that faculty AI profiles and development needs are heterogeneous (Mah and Groß, 2024) and with recent calls to consider the situated dynamics of AI-teacher collaboration (Han et al., 2025).

### 5.4 Limitations and future research

The present findings should be understood within the theoretical and contextual boundaries of the study. The three-wave design provides a longitudinal view of the proposed relationships, although it remains observational, and the focal constructs were assessed by self-report. Future work could complement this evidence with experimental, behavioral, or multi-source approaches. The proposed model focuses on one pathway through which human–AI role ambiguity may relate to professional identity, with meaningful work serving as an intermediate mechanism. Professional identity, however, is shaped by a broader set of experiences and institutional conditions. Future research could therefore extend the model by considering additional mechanisms, such as autonomy, professional recognition, self-efficacy, or perceived replacement risk, and by examining whether several mechanisms operate in parallel.

The meaning of human–AI role ambiguity may also develop as AI becomes more deeply embedded in academic work. At present, uncertainty may center on task allocation, authorship, verification, and accountability, but these boundaries are likely to evolve with changes in technology, institutional policy, and professional norms. Future studies could examine whether different forms of ambiguity emerge as human–AI collaboration becomes more routine and whether their implications differ across teaching, research, supervision, and administrative work.

The findings are also situated in the context of university faculty in mainland China. Professional identity and expectations about responsibility, authority, and technology use are partly shaped by institutional and cultural environments. Replication across educational systems, disciplines, career stages, and organizational settings would help clarify which relationships are broadly applicable and which are context dependent.

In conclusion, the study suggests that the professional implications of GenAI in higher education depend not only on whether teachers use AI, but also on whether the human role remains intelligible. Clearer allocation of tasks, decision rights, authorship, verification, and accountability may help preserve meaningful professional contribution as academic work becomes increasingly AI-supported.

## Data Availability

The raw data supporting the conclusions of this article will be made available by the authors, without undue reservation.
